# Persistent peri-device leak following left atrial appendage occlusion treated with hybrid coil- and plug-based closure: a case report

**DOI:** 10.1093/ehjcr/ytag817

**Published:** 2026-10-31

**Authors:** Ardashir Ibrahim Sulaiman, Jens Trøan, Martin Kirk Christensen, Lisette Okkels Jensen, Mikkel Hougaard

**Affiliations:** Department of Cardiology, Odense University Hospital, J.B. Winsløwsvej 4, Odense C 5000, Denmark; Department of Cardiology, Odense University Hospital, J.B. Winsløwsvej 4, Odense C 5000, Denmark; Department of Cardiology, Odense University Hospital, J.B. Winsløwsvej 4, Odense C 5000, Denmark; Department of Cardiology, Odense University Hospital, J.B. Winsløwsvej 4, Odense C 5000, Denmark; Department of Cardiology, Odense University Hospital, J.B. Winsløwsvej 4, Odense C 5000, Denmark

**Keywords:** Left atrial appendage occlusion, Peri-device leak, Coil embolization, Vascular plug, Atrial fibrillation, Case report

## Abstract

**Background:**

Left atrial appendage occlusion (LAAO) is an emerging alternative to long-term anticoagulation in patients with atrial fibrillation, but peri-device leaks remain a clinical challenge.

**Case summary:**

A 63-year-old man with permanent atrial fibrillation, heart failure, a prior ischaemic stroke history was referred for LAAO due to recurrent anticoagulation-related bleeding. Pre-procedural computer tomography (CT) showed a markedly enlarged appendage suitable for a 35-mm WATCHMAN FLX, which was implanted under intracardiac echocardiography guidance, with subsequent transition to aspirin. Three months later, CT revealed a tilted device with a peri-skirt leak; an ischaemic stroke had occurred in the interim, and anticoagulation was resumed. An Amplatzer Vascular Plug II was implanted but follow-up CT demonstrated persistent contrast passage and more but incomplete occlusion. Three months later, the patient experienced a second stroke, and CT confirmed ongoing peri-device flow. A hybrid leak-closure procedure was performed with coils in the appendage and within the device, followed by a 12-mm plug at the leak channel, achieving near-complete occlusion. At 7-month follow-up, the patient remained on rivaroxaban therapy, briefly interrupted due to haematuria associated with nephrolithiasis.

**Discussion:**

Most cases of peri-device leak after LAAO are related to suboptimal device alignment, incomplete endothelialization, or persistent peri-skirt channels. In our case, device tilt with a peri-skirt tract persisted despite initial plug-based closure. This case provides insight into the potential role of hybrid coil- and plug-based strategies in enhancing occlusion but also highlights that anticoagulation remains necessary when full occlusion is not obtained.

Learning points

- Left atrial appendage occlusion offers an alternative for patients with atrial fibrillation, yet peri-device leaks remain a recognized clinical challenge.
- Hybrid closure strategies combining plug- and coil-based techniques may offer improved occlusion in complex peri-device leaks refractory to single-modality approaches.
- Anticoagulation may remain necessary when PDL persists despite multimodal closure attempts.

## Introduction

Left atrial appendage occlusion (LAAO) has emerged as an alternative to oral anticoagulants (OACs) for patients with nonvalvular atrial fibrillation (NVAF) who are not ideal candidates for sustained anticoagulation.^1,2^ This includes patients with contraindications to long-term OAC or recurrent clinically significant bleeding during anticoagulation. LAAO currently holds a Class IIb recommendation in the ESC guidelines.^3^

Although LAAO has demonstrated effective stroke prevention in selected populations, peri-device leaks (PDLs) remain a recognized limitation.^4–6^

In this case report, we described a persistent PDL despite prior percutaneous closure attempt complicated by recurrent ischaemic strokes.

## Summary figure

**Figure:**
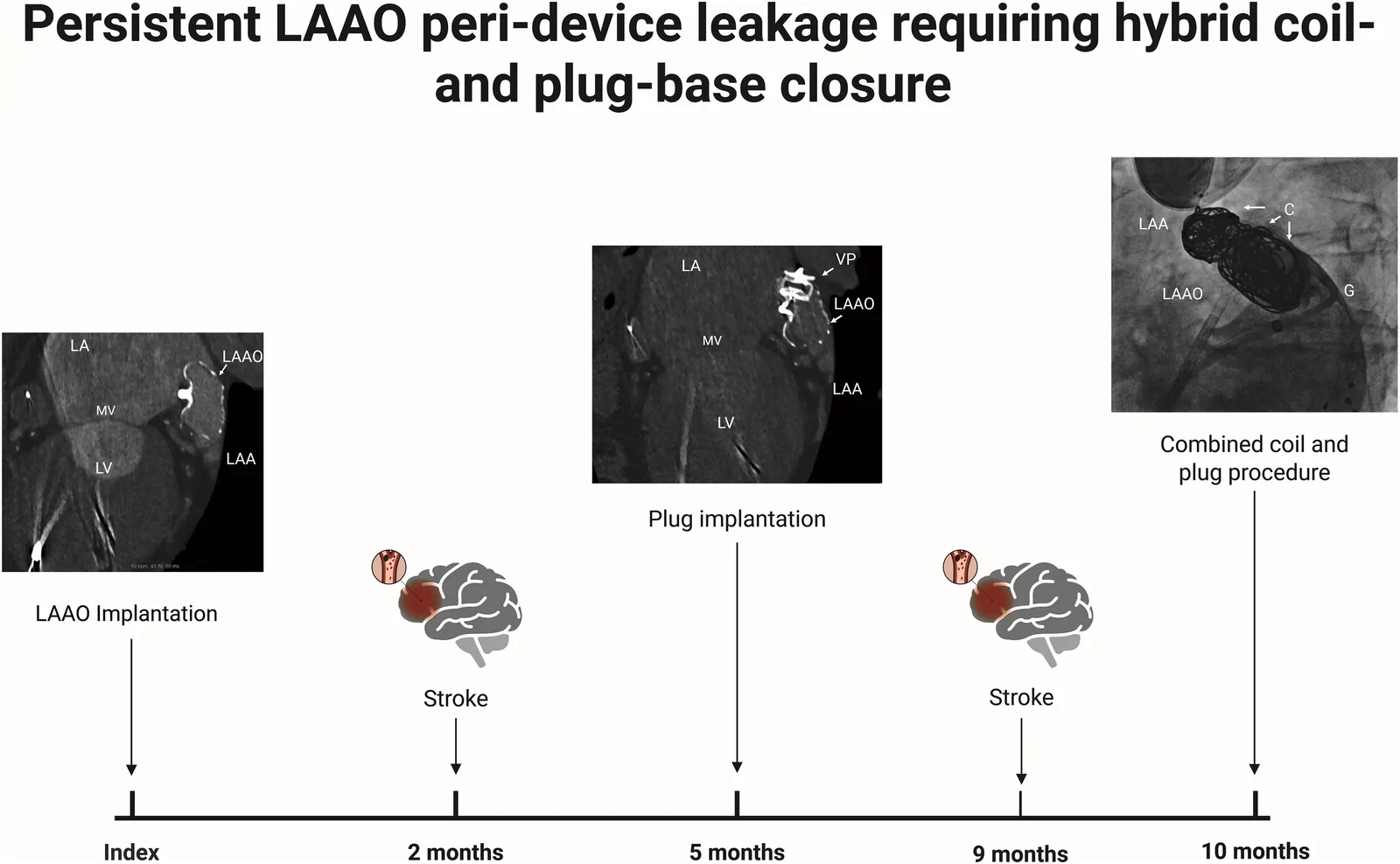


## Case presentation

A 63-year-old man with permanent non-valvular AF (NVAF) was referred for LAAO implantation due to recurrent anticoagulation-related bleeding. His past medical history included hypertension, a non-ischaemic cardiomyopathy with a left ventricular ejection fraction (LVEF) of 35%, a previous ischaemic stroke while on anticoagulation, multiple prior AF ablations, and His-bundle ablation with a biventricular defibrillator device implantation. The patient’s HAS-BLED score was 2, and he had experienced persistent haematuria on rivaroxaban, apixaban, and warfarin, making treatment with OACs challenging. Other causes of haematuria, including infection, renal lithiasis, and malignancy, had been excluded.

Pre-procedural cardiac computer tomography (CT) demonstrated a markedly enlarged left atrial appendage (LAA) suitable for a 35-mm WATCHMAN FLX (Boston Scientific, Marlborough, MA, USA) device. The patient underwent LAAO device implantation under intracardiac echocardiography (ICE) guidance. The device was implanted with satisfactory positioning, compression, and tug test. The patient was discharged on aspirin therapy.

At 3-month follow-up, cardiac CT revealed a tilted device with a 4.3-mm peri-skirt leak and contrast passage into the LAA with no device-related thrombus (*Figure 1A*). In the interim, the patient had been admitted with a non-disabling ischaemic stroke. Carotid ultrasonography showed no carotid stenosis, while transoesophageal echocardiography (TEE) confirmed device malalignment and turbulent flow behind the device skirt (*Figures 1B* and  *2A*), after which anticoagulation with rivaroxaban was reinitiated.

**Figure 1:**
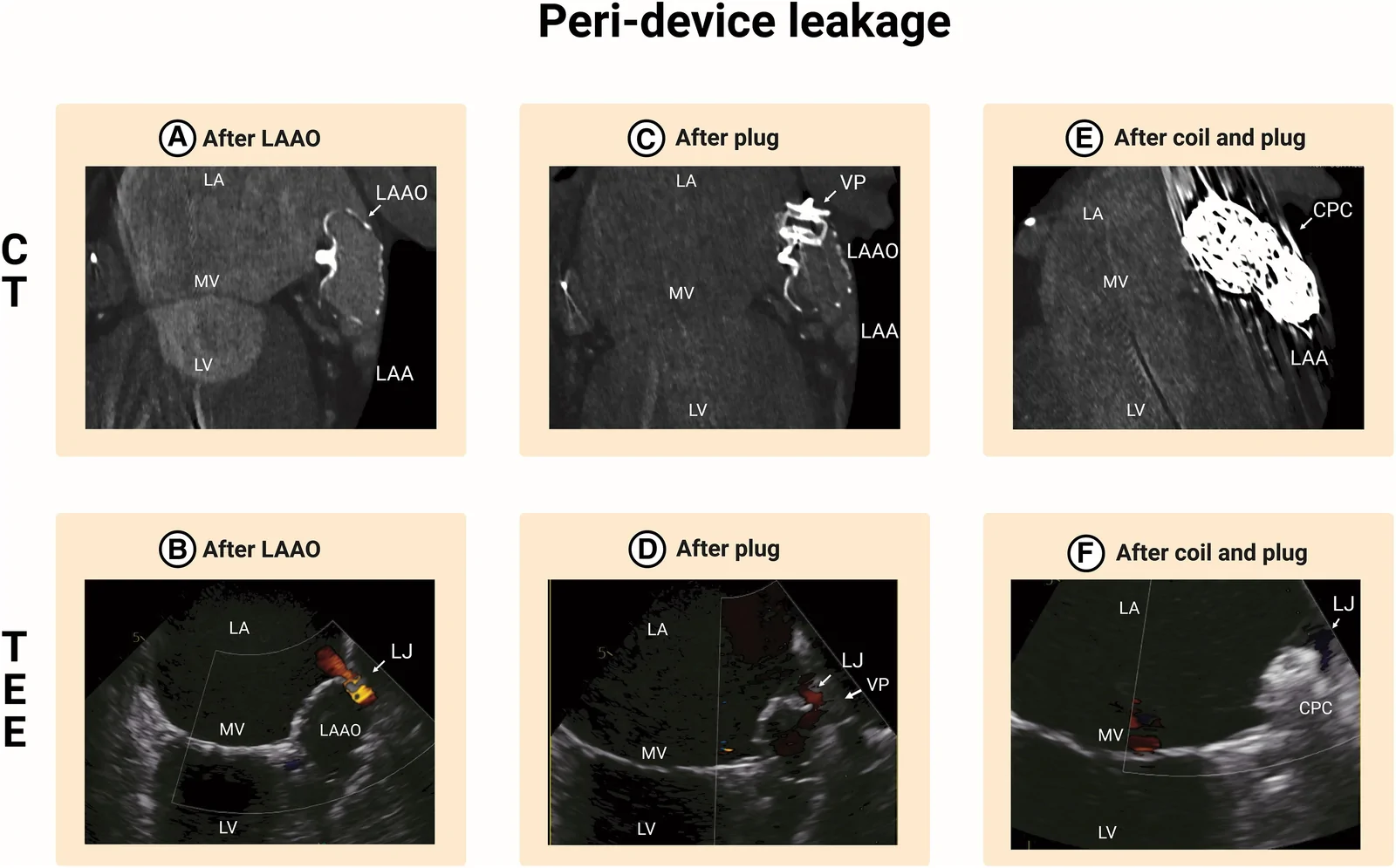
Cardiac computer tomography demonstrates a peri-device leak and incomplete occlusion of the left atrial appendage after left atrial appendage occluder (*A*); a partial reduction of contrast passage following implantation of a vascular plug (*B*); and the hybrid coil-and-plug complex in the auricle (*C*). Transoesophageal echocardiography demonstrates a prominent colour Doppler jet adjacent to the left atrial appendage occluder device consistent with peri-device leak (*D*); a reduced but still detectable residual flow jet following plug implantation, indicating incomplete occlusion of the auricle (*E*); and near-complete sealing of the peri-device leak after the hybrid closure strategy (*F*). CPC, coil-plug-complex; LA, left atrium; LAA, Left atrial appendage; LJ, leak jet; LV, left ventricle; MV, mitral valve; VP, vascular plug. Created in BioRender. Trøan, J. (2026) https://BioRender.com/4nlynt4.

**Figure 2:**
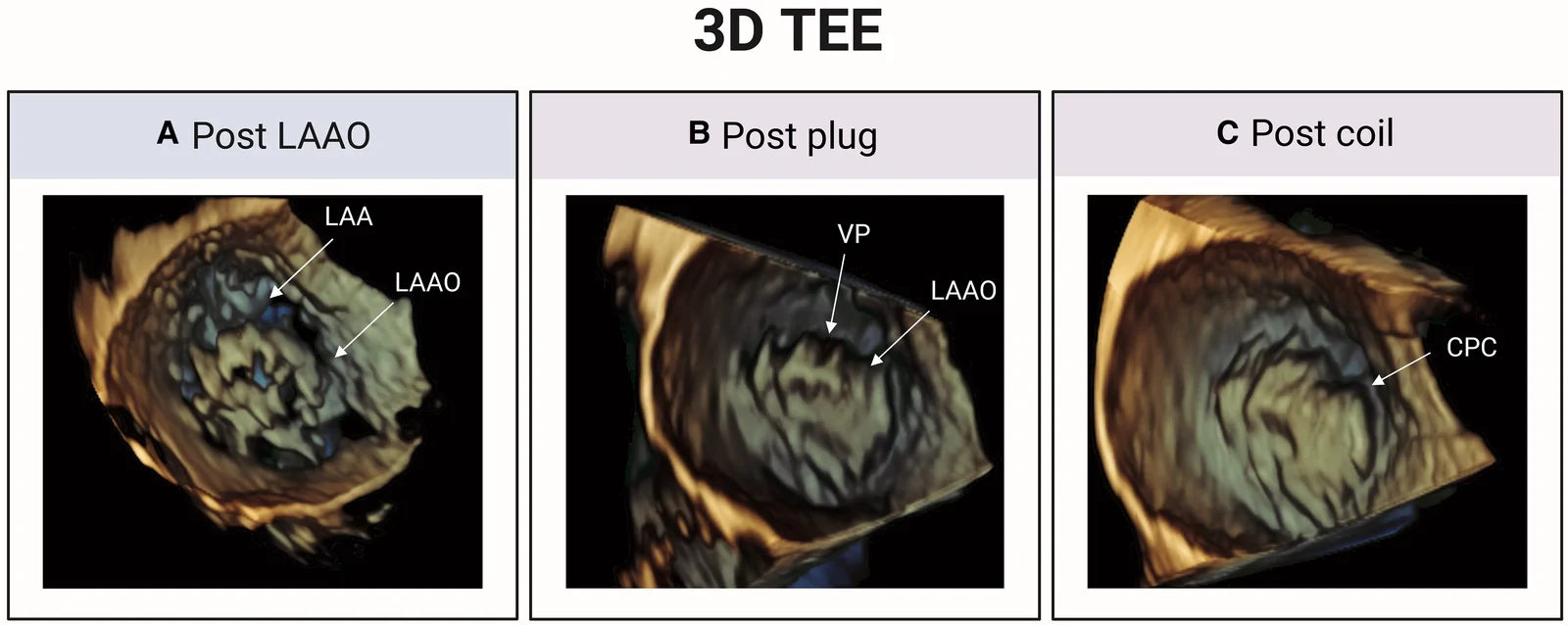
Three-dimensional transoesophageal echocardiography illustrating the left atrial appendage after left atrial appendage occluder (*A*), after plug implantation (*B*), and after the hybrid coil- and-plug procedure (*C*). CPC, coil-plug-complex; LAA, left atrial appendage; VP, vascular plug. Created in BioRender. Trøan, J. (2026) https://BioRender.com/4kz2br8.

Given the persistent leak and the clinical event, the patient was referred for percutaneous leak closure, where a 14-mm Amplatzer Vascular Plug II (AVP2) was implanted into the peri-device cavity. The plug was positioned successfully showing no contrast opacification of the LAA on angiography and no residual insufficiency on TEE. However, the follow-up CT 1 month later showed persistent contrast passage through the plug (*Figure 1C*). To promote closure of the device, rivaroxaban was discontinued and the patient was transitioned to aspirin.

Three months later, the patient suffered a second, likewise non-disabling ischaemic stroke, and rivaroxaban was reinitiated in response. Repeat CT and TEE demonstrated persistent peri-device flow (*Figures 1D* and *2B*). After multidisciplinary discussion, further percutaneous closure using a combined coil-embolization and plug-based strategy was planned.

During the second leak-closure procedure, using a steerable sheath and JR3.5 guiding catheter, a wire was advanced into the LAAO device and subsequently into the distal LAA. Under combined fluoroscopic (*Figure 3A*) and ICE (*Figure 4*) two long coils were deployed into the distal LAA, followed by four coils within the LAAO device (*Figure 3B–C*). Finally, a 12-mm AVP2 was implanted at the entry of the peri-skirt defect (*Figure 3D*). Post-deployment angiography and ICE demonstrated only minimal residual flow and a densely consolidated device-coil complex (*Figure 3E*).

**Figure 3:**
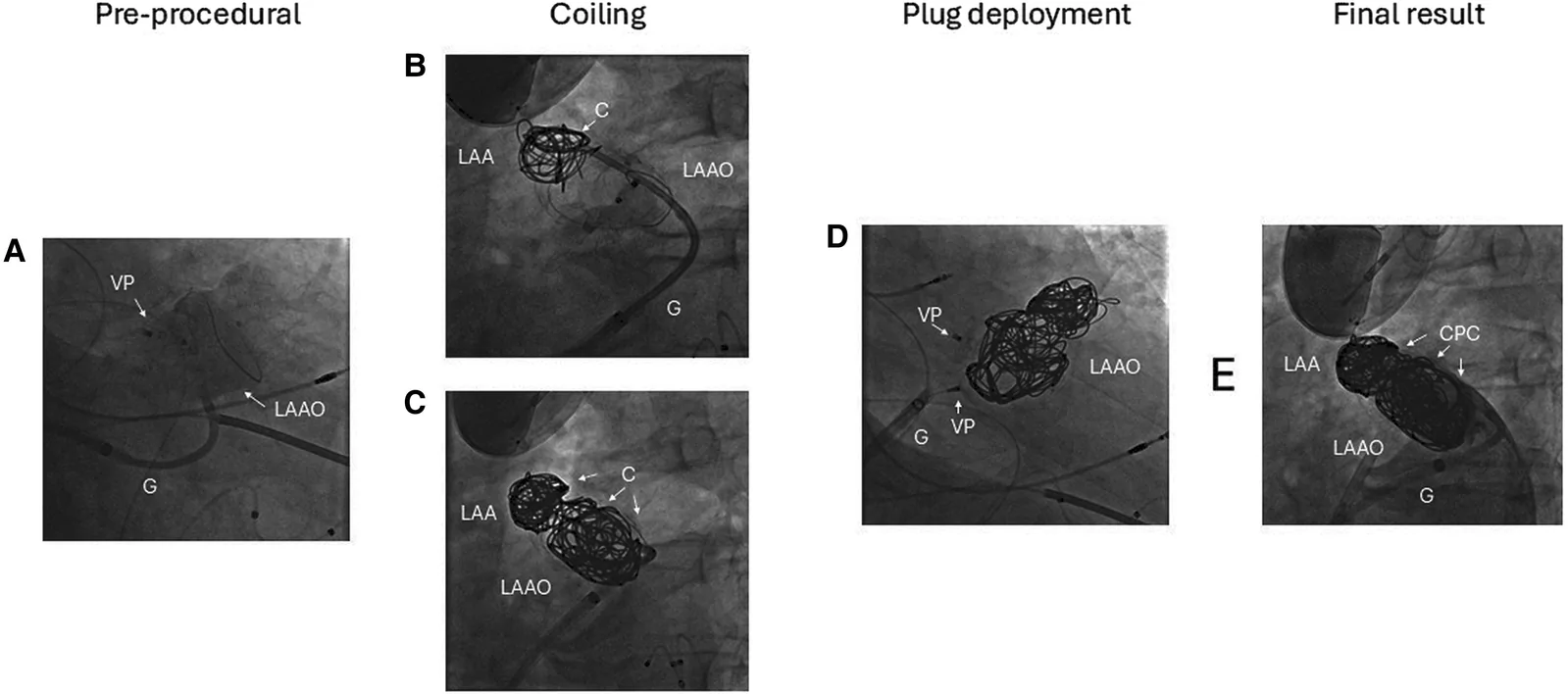
Pre-procedural fluoroscopy showing a persistent peri-skirt leak (*A*), coil deployment in the distal left atrial appendage (*B*) and within the left atrial appendage occluder device (*C*), implantation of a 12-mm AVP2 at the peri-skirt channel (*D*), and final angiographic result demonstrating a densely consolidated coil-device complex with minimal residual flow (*E*). C, coil; CPC, coil-plug-complex; G, guiding catheter; LA, left atrium; VP, vascular plug.

**Figure 4:**
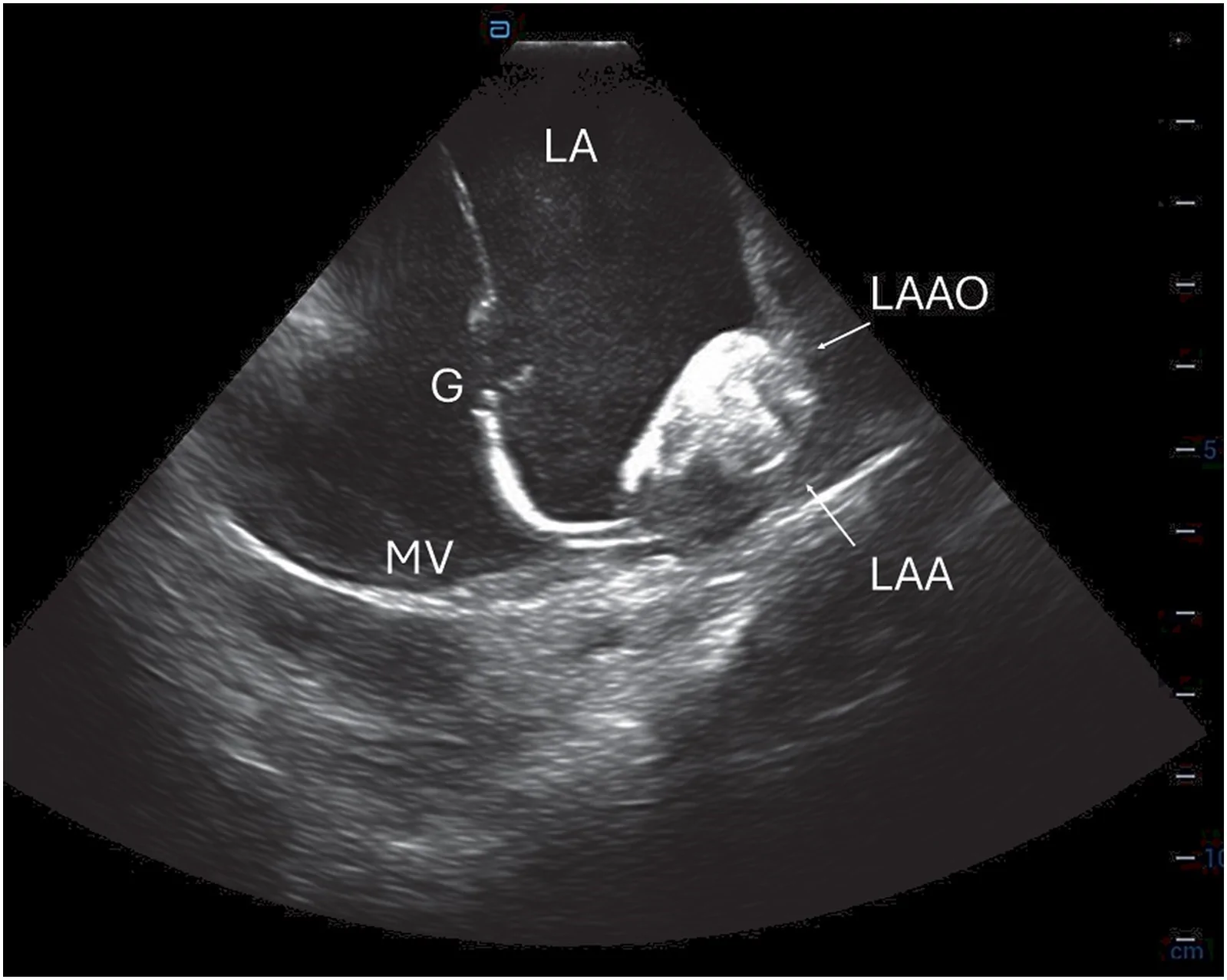
Pre-procedural intracardiac echocardiography before combined coil and plug-based procedure demonstrating the left atrial auricle occlusion device in the left atrial appendage next to the mitral valve and the guiding catheter in the left atrium.

The patient was discharged the following day in stable condition on continued rivaroxaban treatment. Follow-up CT was not of diagnostic quality due to coil artefacts (*Figure 1E*), and a subsequent TEE showed still incomplete thrombosis of the device-coil complex with a minor PDL still present (*Figures 1F* and *2C*). As a result of the recurrent strokes on aspirin therapy and persistent PDL, the patient was continued on rivaroxaban.

During 7-month follow-up, the patient was admitted with haaematuria associated with nephrolithiasis, prompting a 10-day interruption of rivaroxaban. No further ischaemic strokes or bleeding events occurred (*Figure 5*). TEE performed seven months after the hybrid procedure demonstrated an unchanged persistent PDL.

**Figure 5:**
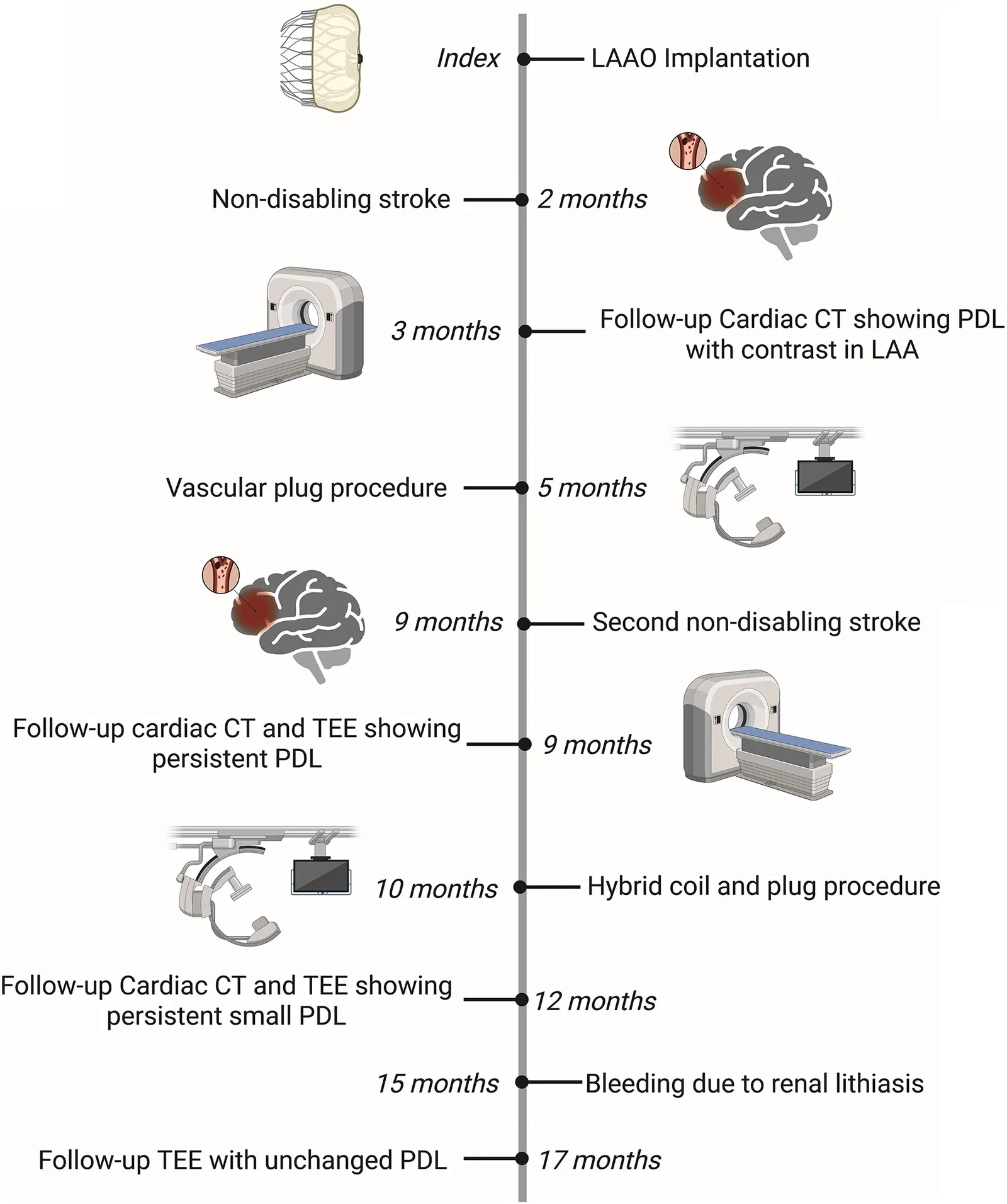
Timeline of the case report. CT, computer tomography; LAA, left atrial appendage, LAAO; left atrial appendage occlude; PDL, peri-device leak; TEE, transoesophageal echocardiography. Created in BioRender. Trøan, J. (2026) https://BioRender.com/3dj0rub.

## Discussion

In clinical practice, successful LAAO depends on achieving complete sealing of the appendage, a process influenced by anatomical conformity, device positioning, and adequate endothelialization.

In our case, device implantation initially appeared satisfactory on angiography, yet a clinically significant PDL was later identified on cardiac CT. The leak likely developed due to device tilt and peri-skirt flow; this tilt may reflect anatomical constraints of the LAA or incomplete seating of the device. From a technical standpoint, operator-dependent factors may have contributed, for instance, subtle malalignment during deployment, especially in a challenging anatomy with an enlarged LAA. Despite adherence to recommended procedural steps, PDL may still occur,^1,7^ being observed in 10.5% of patients with the newer WATCHMAN FLX.^6^

Several patient- and procedure-related risk factors for PDL have been described, such as limited device oversizing, reduced LVEF, device-LAA shape mismatch, prior radiofrequency ablation, and male sex, some of which were present in our patient.^8^ In retrospect, these features may have predisposed to suboptimal device-appendage interaction and subsequent leak.

The clinical implications of PDL are still under investigation. In the initial WATCHMAN device trial, PDL <5 mm was considered benign^7^; however, a subsequent analysis showed that PDL ≤5 mm persisting at 1-year follow-up was associated with an increased risk of thromboembolic events, largely driven by non-disabling stroke, whereas PDL detected at 45 days was not.^9^ Given the patient’s two strokes, anticoagulation remained essential to reduce the risk of further thromboembolic events, even in the presence of only minor residual leakage.

Management of PDL remains challenging, with limited data regarding optimal treatment.^5^ Several treatment strategies may be considered including continued anticoagulation and percutaneous closure using coils, vascular plugs, or additional occluder devices. The choice of strategy should be tailored to the mechanism and morphology of the residual leak.^10,11^

In our case, continued anticoagulation alone was considered an unsatisfactory long-term strategy given the patient's history of recurrent anticoagulation-related bleeding. A plug-based strategy was initially selected to achieve focal mechanical closure of the peri-device cavity. Unfortunately, the initial plug-based closure failed to achieve complete occlusion, possibly due to a shallow and irregular landing zone where the circular plug could not adequately conform. Therefore, an additional plug alone was considered unlikely to achieve durable closure.

Coil embolization was therefore selected to fill the irregular residual cavity and provide a thrombogenic scaffold. However, because a discrete peri-skirt entry channel remained, coil embolization alone was considered unlikely to provide sufficient mechanical sealing at the origin of the leak. A vascular plug was therefore implanted at the entry of the peri-skirt channel to reduce residual inflow and consolidate the coil mass.

Nevertheless, even with more complete sealing, minor persistent PDL may carry clinical significance and thereby necessitate continued anticoagulation.

## Conclusion

In conclusion, this case demonstrates that even a sequential hybrid coil-and-plug approach may fail to achieve complete occlusion when marked device tilt and peri-skirt channel are present. The clinical implication is that anatomical pre-disposing factors may supersede technical strategy, and anticoagulation should not be assumed dispensable after multimodal attempts at PDL closure.

## Lead author biography


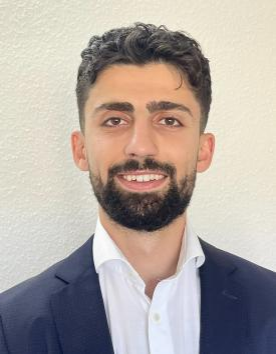
Ardashir Ibrahim Sulaiman is a medical student at the University of Southern Denmark. His academic interests include interventional cardiology and clinical research.

## Data Availability

Data regarding this case is available at reasonable request.

## Notes

**Consent:** Written consent, in line with the COPE guidelines, for the submission and publication of this case was obtained from the patient.

## References

[1] Reed GW, Nakhla S, Miyasaka R, Harb S, Kanj M, Wazni O, et al Left atrial appendage occlusion: expanding indications and new developments. Struct Heart 2025;9:100354. 10.1016/j.shj.2024.100354 PMC11864148 40017832

[2] Holmes DR, Jr., Alkhouli M, Reddy V. Left atrial appendage occlusion for the unmet clinical needs of stroke prevention in nonvalvular atrial fibrillation. Mayo Clin Proc 2019;94:864–874. 10.1016/j.mayocp.2018.09.025 30962008

[3] Van Gelder IC, Rienstra M, Bunting KV, Casado-Arroyo R, Caso V, Crijns HJGM, et al 2024 ESC Guidelines for the management of atrial fibrillation developed in collaboration with the European Association for Cardio-Thoracic Surgery (EACTS): developed by the task force for the management of atrial fibrillation of the European Society of Cardiology (ESC), with the special contribution of the European Heart Rhythm Association (EHRA) of the ESC. Endorsed by the European Stroke Organisation (ESO). Eur Heart J 2024;45:3314–3414. 10.1093/eurheartj/ehae176 39210723

[4] Alkhouli M, De Backer O, Ellis CR, Nielsen-Kudsk JE, Sievert H, Natale A, et al Peridevice leak after left atrial appendage occlusion: incidence, mechanisms, clinical impact, and management. JACC Cardiovasc Interv 2023;16:627–642. 10.1016/j.jcin.2022.12.006 36990553

[5] Saw J, Holmes DR, Cavalcante JL, Freeman JV, Goldsweig AM, Kavinsky CJ, et al SCAI/HRS expert consensus statement on transcatheter left atrial appendage closure. Heart Rhythm 2023;20:e1–e16. 10.1016/j.hrthm.2023.01.007 36990925

[6] Kar S, Doshi SK, Sadhu A, Horton R, Osorio J, Ellis C, et al Primary outcome evaluation of a next-generation left atrial appendage closure device: results from the PINNACLE FLX trial. Circulation 2021;143:1754–1762. 10.1161/CIRCULATIONAHA.120.050117 33820423

[7] Viles-Gonzalez JF, Kar S, Douglas P, Dukkipati S, Feldman T, Horton R, et al The clinical impact of incomplete left atrial appendage closure with the Watchman Device in patients with atrial fibrillation: a PROTECT AF (percutaneous closure of the left atrial appendage versus warfarin therapy for prevention of stroke in patients with atrial fibrillation) substudy. J Am Coll Cardiol 2012;59:923–929. 10.1016/j.jacc.2011.11.028 22381428

[8] Helal B, Khan J, AlJayar D, Khan MS, Alabdaljabar MS, Asad ZUA, et al Risk factors, clinical implications, and management of peridevice leak following left atrial appendage closure: a systematic review. J Interv Card Electrophysiol 2024;67:865–885. 10.1007/s10840-023-01729-z 38182966

[9] Dukkipati SR, Holmes DR, Jr., Doshi SK, Kar S, Singh SM, Gibson D, et al Impact of peridevice leak on 5-year outcomes after left atrial appendage closure. J Am Coll Cardiol 2022;80:469–483. 10.1016/j.jacc.2022.04.062 35902169

[10] Della Rocca DG, Horton RP, Di Biase L, Bassiouny M, Al-Ahmad A, Mohanty S, et al First experience of transcatheter leak occlusion with detachable coils following left atrial appendage closure. JACC Cardiovasc Interv 2020;13:306–319. 10.1016/j.jcin.2019.10.022 31954677

[11] Piayda K, Sievert K, Della Rocca DG, Adeola OG, Alkhouli M, Yoo D, et al Safety and feasibility of peri-device leakage closure after LAAO: an international, multicentre collaborative study. EuroIntervention 2021;17:e1033–e1040. 10.4244/EIJ-D-21-00286 PMC9724933 34219662

